# Miglustat in Neuronopathic Lysosomal Storage Disorders: Biological Rationale, Clinical Evidence, and Limits of Repurposing

**DOI:** 10.3390/ijms27188336

**Published:** 2026-09-19

**Authors:** Patryk Lipiński, Grzegorz Węgrzyn

**Affiliations:** 1 Third Department of Pediatrics, Center of Postgraduate Medical Education, 05-092 Dziekanów Leśny, Poland; plipinski@cmkp.edu.pl; 2 Department of Molecular Biology, Faculty of Biology, University of Gdańsk, Wita Stwosza 59, 80-308 Gdańsk, Poland

**Keywords:** miglustat, lysosomal storage disorders, substrate reduction therapy, glycosphingolipids, neurodegeneration, Niemann–Pick disease type C, gangliosidosis, neuronal ceroid lipofuscinosis, blood–brain barrier, drug repurposing

## Abstract

Neuronopathic lysosomal storage disorders remain difficult to treat because conventional enzyme replacement therapies generally do not reach therapeutically meaningful concentrations in the central nervous system. Miglustat (1,5-(butylimino)-1,5-dideoxy-D-glucitol; N-butyl-deoxynojirimycin) is an orally administered iminosugar that crosses the blood–brain barrier and reduces glycosphingolipid synthesis through inhibition of glucosylceramide synthase. This narrative review evaluated the biological rationale, clinical evidence, and limitations of miglustat repurposing across neuronopathic lysosomal storage disorders. PubMed/MEDLINE was searched from inception through 31 July 2026, with backward citation searching. Miglustat has established clinical benefit in Niemann–Pick disease type C, whereas a randomized trial in Gaucher disease type 3 did not demonstrate neurological efficacy. Evidence in GM1 gangliosidosis, Sandhoff disease, mucolipidosis type IV, and *CLN3* disease is encouraging but limited to preclinical studies, small uncontrolled series, or individual reports; controlled studies in late-onset Tay–Sachs disease and mucopolysaccharidosis type III were negative. Central nervous system penetration and biochemical target engagement therefore cannot be equated with clinical disease modification. Future repurposing should require disease-specific mechanistic justification, pharmacodynamic biomarkers, natural-history comparators, and intervention before irreversible neurodegeneration.

## 1. Introduction

Lysosomal storage disorders comprise a large and increasingly heterogeneous group of inherited diseases caused by defects in lysosomal hydrolases, membrane proteins, transporters, trafficking proteins, and other components required for lysosomal homeostasis. Neurological involvement occurs in a substantial proportion of these disorders and remains one of their most difficult therapeutic manifestations. Although enzyme replacement therapy (ERT) has transformed the management of several systemic lysosomal storage disorders (LSDs), intravenously administered lysosomal enzymes generally fail to cross the blood–brain barrier (BBB) in therapeutically meaningful amounts. Consequently, successful correction of visceral disease does not necessarily prevent progressive neurological deterioration [1,2,3].

Small molecules capable of reaching the central nervous system (CNS) represent an attractive therapeutic strategy. Miglustat (1,5-(butylimino)-1,5-dideoxy-D-glucitol or N-butyl-deoxynojirimycin; NB-DNJ) is an orally administered iminosugar that inhibits UDP-glucose:ceramide glucosyltransferase, commonly referred to as glucosylceramide synthase (GCS). GCS catalyzes the formation of glucosylceramide from ceramide and UDP-glucose and represents the first committed step in the biosynthesis of more complex glycosphingolipids (GSLs). Inhibition of this pathway reduces metabolic flux toward lactosylceramide and downstream gangliosides and other complex GSLs [1,2].

This mechanism provided the basis for substrate reduction therapy (SRT). In contrast to ERT, SRT does not restore the deficient lysosomal enzyme but reduces the rate at which substrate is generated. The classical concept assumes that lowering substrate production may allow residual lysosomal degradative activity to establish a new metabolic equilibrium and thereby reduce or delay progressive storage. Miglustat was initially developed within this framework and became the first SRT approved for Gaucher disease type 1 in patients unsuitable for ERT [3].

Miglustat is particularly interesting in the context of neuronopathic LSDs because it enters the CNS. Human pharmacokinetic studies have demonstrated measurable concentrations in cerebrospinal fluid (CSF), with steady-state CSF concentrations in patients with Gaucher disease type 3 reaching approximately 31–67% of corresponding plasma concentrations [2,4]. Its subsequent clinical development in Niemann–Pick disease type C (NPC) provided proof of principle that systemic modulation of GSL metabolism can influence neurological disease [3,5].

However, experience that accumulated over more than two decades has demonstrated that CNS penetration alone does not predict neurological efficacy. Miglustat has been investigated in disorders characterized by primary GSL storage, secondary abnormalities of GSL metabolism, and more complex disturbances of lysosomal lipid homeostasis, with outcomes ranging from controlled evidence consistent with neurological benefit to unequivocally negative clinical trials. These observations make miglustat an unusually informative model for addressing a broader translational question: under what biological and pharmacological circumstances can manipulation of glycosphingolipid metabolism modify lysosomal neurodegeneration? [1,6].

## 2. Scope and Approach

**Figure 1:**
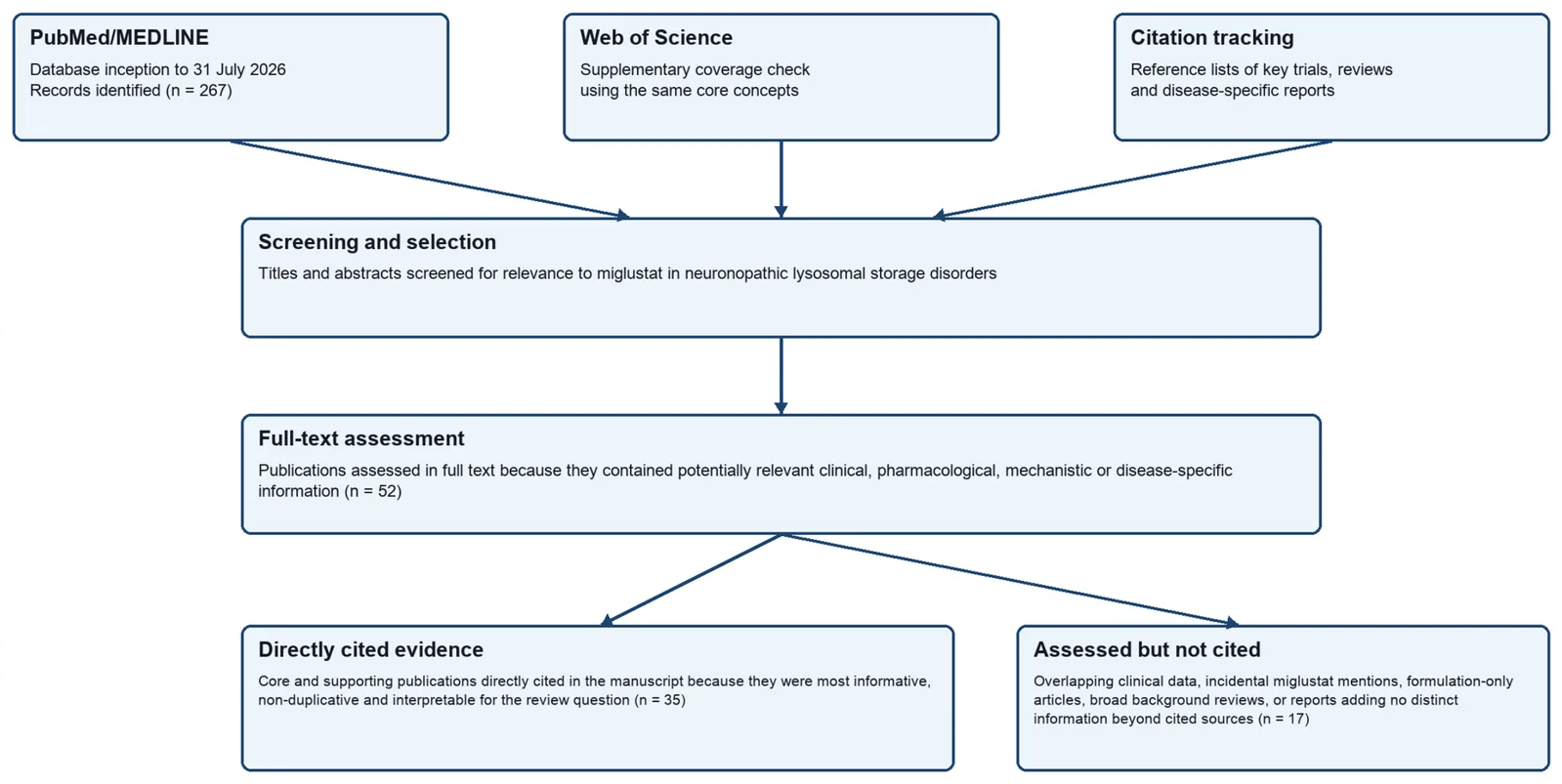
The scheme of the process of literature selection, demonstrating the search parameters and strategies used for literature search and evaluation, as well as indicating the obtained results in this flowchart.

To identify relevant literature for this narrative review, PubMed/MEDLINE (https://pubmed.ncbi.nlm.nih.gov/ last accessed on 31 July 2026) and Web of Science (https://access.clarivate.com/ last accessed on 31 July 2026) were searched from database inception. The core strategy combined “miglustat” or “N-butyldeoxynojirimycin” with terms for neuronopathic lysosomal storage disorders. Because the literature is disease-specific and uses heterogeneous terminology, the search was supplemented with disease and pathway terms, including “Niemann–Pick disease type C”, “Gaucher disease”, “GM1 gangliosidosis”, “GM2 gangliosidosis”, “Tay–Sachs disease”, “Sandhoff disease”, “mucopolysaccharidosis”, “mucolipidosis IV”, “neuronal ceroid lipofuscinosis”, “substrate reduction therapy”, and “glycosphingolipid”. Reference lists of key clinical trials, reviews and recent disease-specific publications were also screened. The PubMed/MEDLINE search identified 267 records. Web of Science and citation tracking were used as supplementary coverage checks to reduce the risk of missing relevant reports, rather than as independent sources for a formal systematic-review count. Titles and abstracts were screened for direct relevance to miglustat in neuronopathic lysosomal storage disorders. Fifty-two publications were assessed in full text because they contained potentially relevant clinical, safety, pharmacokinetic, pharmacodynamic, mechanistic, or disease-specific preclinical information. Of these, 35 publications were directly cited in the final manuscript because they provided the most informative, non-duplicative and interpretable evidence for the review question. The remaining 17 publications were not cited because they contained overlapping clinical data, mentioned miglustat only incidentally, addressed pharmaceutical formulation or broad lysosomal biology without clear relevance to the review question, or did not add distinct information beyond the cited sources. The literature-selection process is summarized in Figure 1.

## 3. Pharmacological Rationale for Miglustat in Neuronopathic Lysosomal Storage Disorders

**Figure 2:**
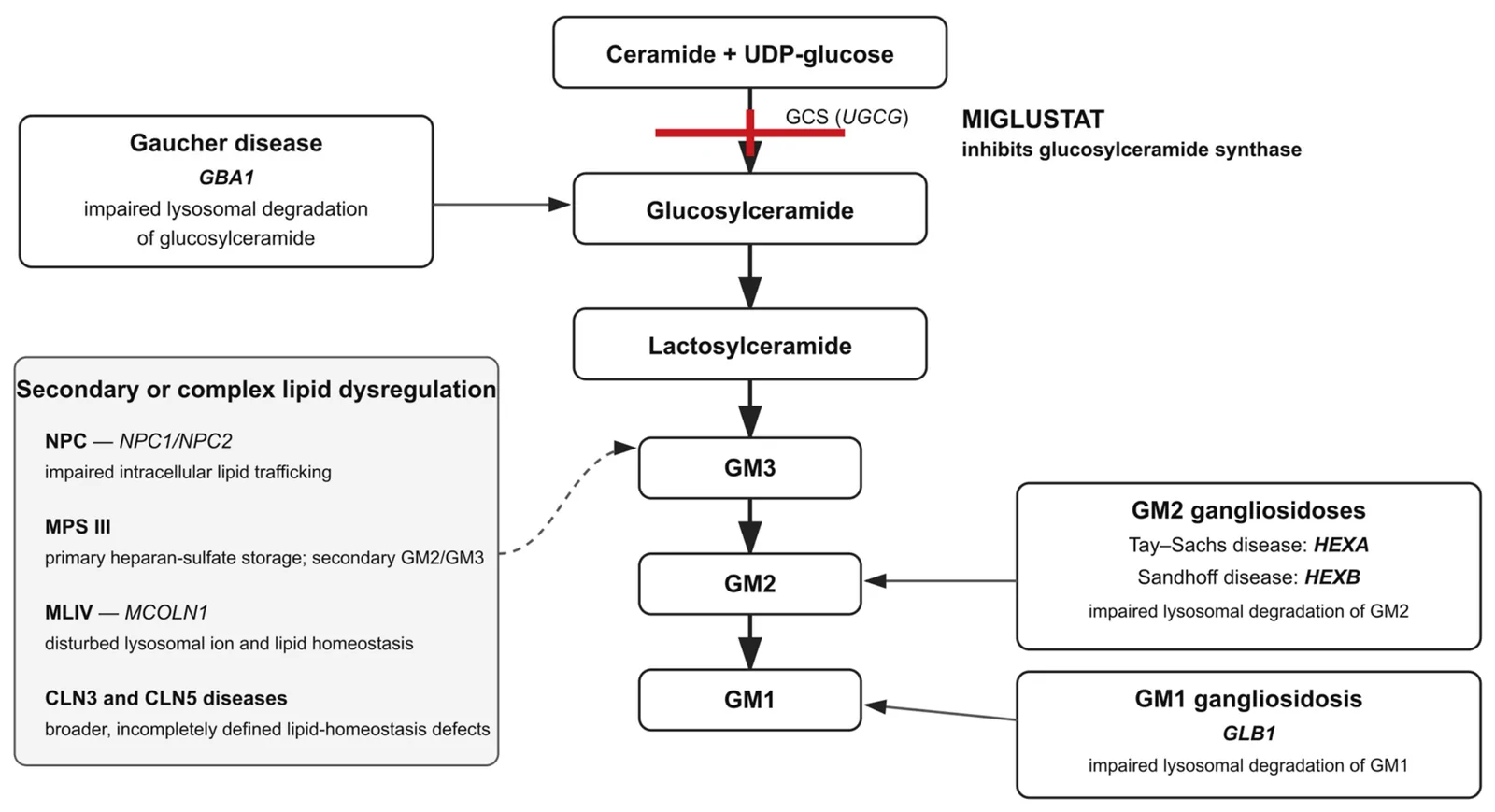
Glycosphingolipid pathway and the stage of action of miglustat. Miglustat inhibits UDP-glucose:ceramide glucosyltransferase, commonly termed glucosylceramide synthase (GCS), thereby reducing the formation of glucosylceramide and downstream glycosphingolipids (the inhibition of the reaction (makred by red arrow) by miglustat is indicated by a horizontal red line). In Gaucher disease and GM1 and GM2 gangliosidoses, the primary molecular defect directly affects glycosphingolipid degradation. In Niemann–Pick disease type C, mucopolysaccharidosis type III, mucolipidosis type IV, and CLN3 and CLN5 diseases, glycosphingolipid abnormalities are secondary or form part of broader disturbances of lysosomal lipid homeostasis. Biochemical proximity to GCS does not by itself predict neurological response to miglustat. Abbreviations: GBA1, β-glucocerebrosidase; GCS, glucosylceramide synthase; GLB1, β-galactosidase; HEXA, β-hexosaminidase subunit α; HEXB, β-hexosaminidase subunit β; MLIV, mucolipidosis type IV; MPS III, mucopolysaccharidosis type III; NPC, Niemann–Pick disease type C; UDP, uridine diphosphate.

Figure 2 summarizes the position of GCS inhibition within glycosphingolipid biosynthesis and its relationship to the primary or secondary lipid abnormalities in the disorders discussed below.

The central position of glucosylceramide within GSL biosynthesis provides a theoretically powerful point of metabolic intervention. Reduction in glucosylceramide synthesis decreases the generation of downstream GSLs, including GM1 and GM2 gangliosides. This mechanism is particularly intuitive in disorders in which the primary lysosomal defect directly prevents degradation of a GSL. In such diseases, decreasing substrate synthesis should reduce the amount of material delivered to an impaired lysosomal degradative pathway [1,2].

Nevertheless, this simple substrate-balance model has important limitations. Its efficacy depends not only on the degree of substrate reduction but also on residual catabolic capacity. In severe infantile disorders associated with almost complete loss of enzyme activity, even substantial reductions in substrate production may be insufficient to restore metabolic equilibrium. Conversely, patients with later-onset phenotypes and measurable residual enzyme activity may theoretically have a greater capacity to benefit from partial substrate reduction. This concept is potentially relevant to the apparently more encouraging observations in juvenile and adult GM1 gangliosidosis compared with rapidly progressive infantile disease [7,8].

CNS exposure represents another critical determinant. Miglustat crosses the BBB, but CSF concentration is only a surrogate for pharmacologically relevant exposure within the brain. It does not establish intracellular drug concentrations within vulnerable neuronal populations, regional distribution, the magnitude of GCS inhibition, or the extent to which synthesis of disease-relevant GSLs is suppressed. Thus, the clinically relevant question is not simply whether miglustat reaches the CNS, but whether tolerable systemic dosing produces sufficient and sustained target engagement in disease-relevant neural cells [4,9].

**Figure 3:**
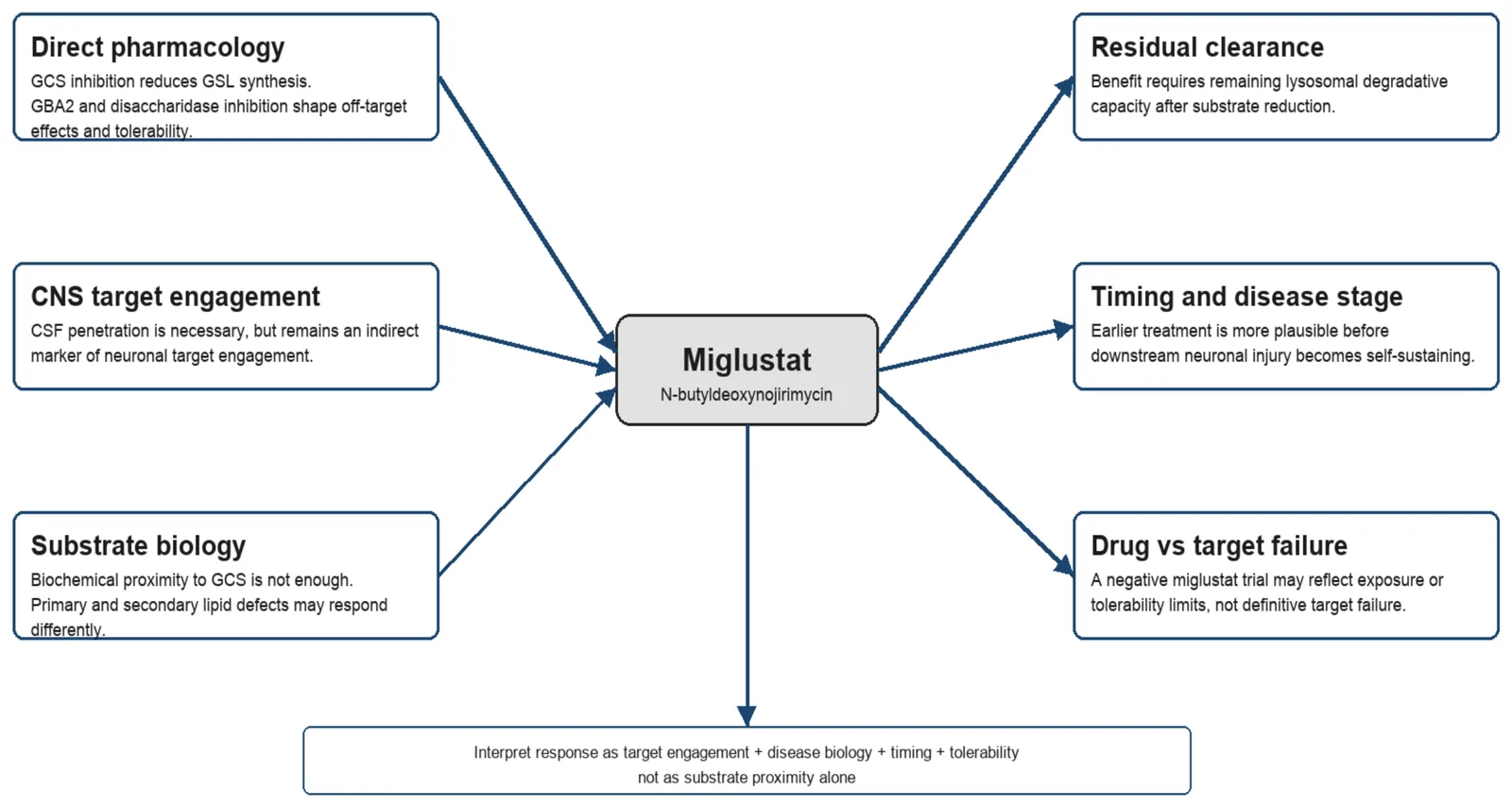
Pharmacological mechanisms and determinants of miglustat response in neuronopathic lysosomal storage disorders. GCS, glucosylceramide synthase; GBA2, non-lysosomal beta-glucosidase 2 encoded by GBA2; CNS, central nervous system; CSF, cerebrospinal fluid.

The pharmacology of miglustat is also more complex than selective GCS inhibition. Miglustat also inhibits non-lysosomal beta-glucosidase 2, the enzyme encoded by GBA2, which is involved in extralysosomal glucosylceramide metabolism [10]. It also inhibits intestinal disaccharidases, which explains common gastrointestinal adverse effects and may limit dose escalation or long-term adherence [11,12]. These off-target effects are clinically relevant because they influence both tolerability and interpretation of response. In addition, a reduction in substrate synthesis can only be useful when there is enough residual lysosomal degradative capacity to restore a more favorable balance between synthesis and clearance. Advanced neurodegeneration, severe infantile phenotypes and late initiation after substantial neuronal loss may therefore blunt the clinical effect even when the biochemical rationale is strong. For future repurposing studies, evidence of CNS exposure should be complemented by pharmacodynamic biomarkers, preferably including CSF or tissue-relevant lipidomic markers, and by endpoints matched to the tempo of the disease. The main pharmacological mechanisms and determinants of response are summarized in Figure 3.

## 4. Niemann–Pick Disease Type C: The Positive Clinical Benchmark

NPC represents the strongest evidence that miglustat can modify the neurological course of a lysosomal disorder. The disease is caused predominantly by pathogenic variants in NPC1, and less frequently NPC2, resulting in impaired intracellular lipid trafficking and complex abnormalities involving unesterified cholesterol, sphingolipids, and GSLs. Importantly, GSL accumulation in NPC is secondary to the trafficking defect rather than the consequence of deficiency of a GSL-degrading hydrolase [13,14,15].

The pivotal randomized controlled study by Patterson et al. included 29 patients aged 12 years or older, of whom 20 received miglustat 200 mg three times daily and nine received standard care for 12 months. A parallel pediatric cohort of 12 younger patients received body-surface-area-adjusted treatment [13]. Horizontal saccadic eye movement velocity was used as an objective neurological outcome because saccadic abnormalities represent a characteristic manifestation of NPC. Treatment was associated with a favorable effect on horizontal saccadic eye movement velocity, with statistical significance in the prespecified analysis excluding patients receiving benzodiazepines. Other neurological outcomes, including swallowing and ambulatory measures, were generally consistent with stabilization or improvement in subsets of patients [13].

Long-term extension studies subsequently supported sustained neurological stabilization during continued miglustat exposure [14]. In contrast, a French national retrospective study of early-infantile NPC found no significant long-term neurodevelopmental benefit or survival advantage in 10 miglustat-treated patients compared with 16 untreated patients [16]. This phenotype-specific result cautions against extrapolating findings from juvenile and adult cohorts to rapidly progressive early-infantile disease. This is particularly important when interpreting therapeutic efficacy in progressive neurodegenerative diseases. A drug that reduces the rate of decline without restoring previously lost neurological function may still exert a clinically meaningful disease-modifying effect.

Therefore, NPC established two important principles. First, a secondary GSL abnormality can represent a therapeutically relevant target. Second, clinical benefit from SRT does not require the primary molecular defect to reside directly within the GSL degradation pathway. These observations provided much of the rationale for investigating miglustat more broadly across neuronopathic LSDs.

## 5. Neuronopathic Gaucher Disease: Strong Biochemical Rationale but Disappointing Neurological Translation

Gaucher disease provides perhaps the most direct biochemical rationale for GCS inhibition. Deficiency of lysosomal β-glucocerebrosidase caused by pathogenic variants in GBA1 results in accumulation of glucosylceramide and related lipids. In Gaucher disease type 3 (GD3), ERT effectively controls many systemic manifestations but has limited effects on neurological disease because recombinant enzyme does not adequately reach the CNS [2,4].

Hence, miglustat appeared particularly attractive as a CNS-directed complement to ERT. Schiffmann et al. evaluated this hypothesis in a 24-month phase II study involving 30 patients with GD3. During the first 12 months, 21 patients were randomized to miglustat in addition to existing therapy and nine continued without miglustat, followed by an optional 12-month extension [4]. Quantitative saccadic eye-movement measurements were among the principal neurological endpoints, supplemented by neurological and neuropsychological assessments. The published trial report did not provide patient-level GBA1 genotypes, so the neurological outcome cannot be stratified by genotype or by residual glucocerebrosidase activity. This is an important limitation, because GD3 cohorts are frequently enriched for severe GBA1 variants, including p.Leu483Pro, historically referred to as L444P, and very low residual enzyme activity may leave insufficient lysosomal catabolic capacity for substrate reduction alone to translate into a measurable neurological benefit.

Despite measurable CNS exposure, the study did not demonstrate a statistically significant effect on the principal neurological manifestations of GD3 [4]. This negative result is particularly informative because the drug reached the CNS, its biochemical target lay immediately upstream of the accumulated lipid, and the theoretical rationale for substrate reduction was exceptionally strong.

Several explanations are possible. The degree of GCS inhibition achievable at tolerable doses may have been insufficient within relevant neuronal populations. Alternatively, neurological disease in GD3 may involve toxic metabolites or downstream processes not adequately controlled by reducing glucosylceramide synthesis. Treatment may have also been initiated after irreversible neuronal injury had already occurred.

GD3 therefore provides an important counterpoint to NPC. The comparison demonstrates that biochemical proximity between the drug target and the stored material is not sufficient to predict neurological efficacy.

## 6. GM1 Gangliosidosis: A Compelling but Unproven Target

GM1 gangliosidosis results from deficiency of lysosomal β-galactosidase caused by pathogenic variants in GLB1, leading to progressive accumulation of GM1 ganglioside, particularly in the CNS. Because GM1 biosynthesis depends on the glucosylceramide pathway, the rationale for GCS inhibition is direct [17,18].

Preclinical studies and experiments in patient-derived cells support biochemical target engagement. Miglustat can reduce GM1 accumulation in cellular systems, demonstrating that the metabolic pathway is pharmacologically modifiable [17].

Human evidence remains limited to small uncontrolled series. Deodato et al. described three patients with juvenile or adult GM1 gangliosidosis treated with miglustat [7]. The patients were treated with a dose of 600 mg/day and were reported to show stabilization or improvement in selected neurological measures. In one patient, the 6 min walking distance increased from 338 to 475 m, while other observations included improved movement and speech control [7].

Fischetto et al. subsequently reported four pediatric patients with type 2 GM1 gangliosidosis treated with miglustat [8]. Stabilization or slowing down of neurological progression was reported in several patients, with the more encouraging observations occurring in juvenile phenotypes and in patients treated relatively early.

These reports are intriguing because they are compatible with the theoretical importance of residual β-galactosidase activity. SRT may be more effective when substrate synthesis is reduced in the presence of sufficient residual catabolic activity to process the remaining substrate load. However, GM1 has a heterogeneous natural history, particularly in later-onset forms, and uncontrolled observations cannot distinguish treatment effects from natural variability.

GM1 should therefore be regarded as one of the most biologically compelling unproven applications of miglustat. Existing data justify further investigation but do not establish clinical efficacy.

## 7. GM2 Gangliosidoses: The Translational Paradox

The GM2 gangliosidoses provide perhaps the clearest example of the discrepancy between strong preclinical rationale and disappointing clinical translation. Tay–Sachs disease results from deficiency of β-hexosaminidase A, whereas Sandhoff disease results from deficiency of the β-subunit shared by β-hexosaminidases A and B. Both disorders lead to progressive accumulation of GM2 ganglioside within the CNS [17,18,19,20].

Early experiments in animal models demonstrated that iminosugar-based substrate reduction could delay neurological deterioration and prolong survival [20,21]. These observations provided some of the strongest early evidence supporting CNS-directed SRT.

The clinical experience in late-onset Tay–Sachs disease was considerably less encouraging. Shapiro et al. enrolled 30 adults aged 18–56 years in a randomized study in which 20 patients received miglustat 200 mg three times daily and ten received no miglustat during the initial 12-month phase. This was followed by a 24-month extension during which all participants could receive treatment [22]. Despite the direct relationship between GM2 accumulation and the pathway targeted by miglustat, no significant differences were observed in muscle strength, grip strength, gait, balance, disability, or other efficacy measures. Prolonged exposure during the extension period likewise failed to demonstrate convincing clinical benefit [22].

The Tay–Sachs trial raises a fundamental question. Did GSL synthesis represent the wrong therapeutic target, or did miglustat fail to inhibit the target sufficiently within the human brain? These possibilities cannot be considered equivalent. The negative results of the study established that the tested regimen of miglustat did not provide measurable benefit in symptomatic late-onset Tay–Sachs disease. It does not necessarily mean that more potent and selective brain GCS inhibition would be ineffective.

Sandhoff disease provides related but less definitive human evidence. Strong preclinical effects have been demonstrated in mouse models [20,21], whereas clinical experience is largely anecdotal. Masciullo et al. described a patient with chronic Sandhoff disease treated with miglustat for three years, with apparent stabilization of selected neurological manifestations [23]. However, such a single-patient observation cannot establish efficacy, particularly in a heterogeneous chronic phenotype.

Taken together, the GM2 experience demonstrates that strong target validity and preclinical efficacy do not guarantee successful translation to symptomatic human disease.

## 8. Mucopolysaccharidosis Type III: Secondary Ganglioside Storage as a Therapeutic Target

Mucopolysaccharidosis type III (MPS III, Sanfilippo syndrome) offers a particularly informative test of the hypothesis that secondary GSL abnormalities can drive lysosomal neurodegeneration. The primary metabolic defect involves lysosomal degradation of heparan sulfate rather than GSL catabolism. Nevertheless, affected brains accumulate substantial amounts of secondary GM2 and GM3 gangliosides [22,23,24].

Preclinical findings provided some support for targeting this secondary lipid pathology. In an MPS IIIA mouse model, NB-DNJ improved selected behavioral abnormalities, including innate fear responses and learning [25].

This rationale was subsequently evaluated clinically. Guffon et al. conducted a randomized, double-blind, placebo-controlled study involving 25 children with MPS III [26]. Miglustat did not significantly improve or stabilize the principal behavioral outcomes compared with placebo. Cognitive, sleep, imaging, and other neurological assessments similarly failed to demonstrate convincing benefit [26].

The pharmacodynamic observations were particularly informative. Miglustat entered the CNS, yet treatment did not produce a significant reduction in ganglioside levels [26]. The negative clinical result therefore cannot simply be attributed to failure of BBB penetration. Instead, either the degree of CNS GSL modulation was insufficient or secondary ganglioside accumulation was not sufficiently upstream in the pathogenic cascade to alter disease progression.

The case of MPS III demonstrates why secondary storage should not automatically be considered an actionable therapeutic target. Abnormal lipid accumulation may represent a disease driver, a modifier, or simply a downstream marker of broader lysosomal dysfunction.

## 9. Mucolipidosis Type IV: Experimental Evidence Beyond Classical Glycosphingolipidoses

Mucolipidosis type IV (MLIV) is caused by pathogenic variants in MCOLN1, encoding the lysosomal ion channel TRPML1. The primary molecular defect therefore lies outside the canonical pathway of GSL degradation. Nevertheless, MLIV is associated with profound abnormalities of lysosomal lipid homeostasis [27,28].

Miglustat has shown notable effects in the Mcoln1-deficient mouse model. Treatment initiated early in disease delayed motor deterioration, reduced cerebellar Purkinje-cell loss and microgliosis, and partially corrected abnormal brain GSL profiles [27]. These findings demonstrated that modifying GSL metabolism can influence neurological disease produced by a lysosomal membrane-protein defect.

MLIV is therefore conceptually important even in the absence of human treatment data. It supports the hypothesis that secondary lipid abnormalities may sometimes participate actively in neurodegeneration rather than merely reflecting lysosomal storage.

However, the current evidence remains preclinical, and extrapolation to therapeutic use in patients would be premature.

## 10. *CLN3* Disease: An Emerging Human Signal

*CLN3* disease represents one of the most interesting recent developments in the repurposing of miglustat. *CLN3* disease is a form of neuronal ceroid lipofuscinosis characterized by progressive visual loss, cognitive decline, epilepsy, and motor deterioration. The primary defect involves a lysosomal/endosomal transmembrane protein, and the relationship between *CLN3* dysfunction and GSL metabolism is considerably less direct than in the gangliosidoses [27,28,29,30].

Pietrafusa et al. recently reported long-term miglustat treatment in six patients with genetically confirmed CLN3 disease [30]. Miglustat was administered at the dose up to 15 mg/kg/day or a maximum of 600 mg/day, and patients were evaluated every six months using the Unified Batten Disease Rating Scale (UBDRS). Median follow-up was 3.9 years. The mean annual increase in the UBDRS physical score was 1.96 ± 0.80 points per year, which the investigators considered potentially slower than expected from historical natural-history trajectories [30].

Treatment was generally well tolerated, with predominantly mild and self-limiting gastrointestinal adverse events. Nevertheless, the interpretation of efficacy is constrained by the extremely small sample, absence of randomization, lack of a concurrent control group, and dependence on historical comparisons. Importantly, an accompanying editorial emphasized that the study cannot establish therapeutic efficacy [31].

Additional pediatric observations have described neurological stabilization and possible improvement in visual or adaptive measures during miglustat treatment [32]. These reports remain anecdotal but reinforce the rationale for formal controlled investigation.

*CLN3* disease is particularly important conceptually. If future controlled studies confirm a disease-modifying effect, the finding would suggest that the therapeutic actions of miglustat extend beyond classical substrate reduction in diseases defined by accumulation of a specific GSL. Miglustat might instead be influencing a broader network of lysosomal lipid homeostasis relevant to neuronal survival.

## 11. *CLN5* Disease and the Possible Extension to Other Neuronal Ceroid Lipofuscinoses

*CLN5* disease provides further experimental evidence linking NCL biology to sphingolipid metabolism. Proteomic studies of CLN5-deficient lysosomes identified disturbances in lipid homeostasis and sphingolipid-related pathways [33]. Importantly, miglustat was directly investigated rather than proposed solely on theoretical grounds.

Treatment of CLN5-deficient cellular models influenced disease-associated abnormalities, and miglustat also improved the locomotor phenotype in a cln5-deficient zebrafish model [33]. These observations provide genuine disease-specific preclinical evidence and distinguish CLN5 from other NCL subtypes for which comparable miglustat data are lacking.

However, the findings in *CLN3* and *CLN5* diseases should not be extrapolated to NCLs as a group. The NCLs are genetically and biologically heterogeneous, and the contribution of sphingolipid abnormalities may differ substantially between subtypes. At present, there is insufficient direct evidence to support miglustat repurposing in CLN1, CLN2, CLN6, CLN7, CLN8, and most other NCLs.

The appropriate conclusion is therefore not that miglustat represents a general therapy for NCLs, but that selected NCL subtypes may contain disease-relevant disturbances of lipid homeostasis that warrant targeted investigation.

The disease-specific evidence discussed above is summarized in Table 1.

## 12. Regulatory Status, Tolerability, and Clinical Monitoring

In the European Union and the United Kingdom, miglustat is authorized for progressive neurological manifestations of NPC in adults and children and for selected adults with mild-to-moderate Gaucher disease type 1 for whom enzyme replacement therapy is unsuitable. Regulatory indications differ internationally; in particular, authorization for NPC is not universal. Its use in the other neuronopathic disorders discussed in this review is off-label or investigational [11]. Regulatory authorization for NPC should not be interpreted as evidence of efficacy across all ages and phenotypes.

The most frequent adverse effects of the use of miglustat are gastrointestinal symptoms, particularly diarrhea, flatulence, abdominal pain, reduced appetite, and weight loss, largely related to inhibition of intestinal disaccharidase activity and consequent carbohydrate malabsorption. Individualized reduction in dietary sucrose, lactose, and other carbohydrates, administration between meals, antidiarrheal treatment, or temporary dose reduction may improve tolerability. Tremor is also common and may require dose reduction or discontinuation. Baseline and repeat neurological assessment is advisable because peripheral neuropathy has been reported. Platelet counts should be monitored in NPC, and weight and linear growth should be followed in children. Because miglustat is eliminated predominantly by the kidneys, exposure increases with renal impairment; dose adjustment is required in moderate impairment and use is generally not recommended in severe impairment, according to the applicable product information. Reproductive precautions include avoidance during pregnancy and breastfeeding and attention to the product-specific recommendations concerning male fertility and contraception. Treatment should be initiated and periodically reassessed by clinicians experienced in the relevant lysosomal disorder [11,12].

In summary, recent experience reinforces that miglustat should not be viewed as a uniform class solution for neuronopathic lysosomal storage disorders. Its plausibility differs across diseases according to the stored substrate, the position of GSLs in the disease cascade, the availability of residual enzyme activity, the age at treatment start, and whether meaningful disease-specific endpoints can be captured over a feasible observation period. This explains why NPC remains the main positive clinical benchmark, whereas studies in neuronopathic Gaucher disease, GM1 and GM2 gangliosidoses, MPS III and NCLs have a more cautious disease-by-disease interpretation.

## 13. What Determines Successful Clinical Translation?

Taken together, the available evidence argues against a simple relationship between the biochemical proximity of the stored substrate to GCS and therapeutic response. Indeed, one of the most striking observations is that the apparently most obvious metabolic targets have not necessarily produced the most convincing neurological outcomes.

GD3 and Tay–Sachs disease are characterized by direct relationships between the primary storage abnormality and GSL metabolism, yet controlled studies failed to demonstrate meaningful neurological benefit [4,22]. In contrast, NPC is caused by defective intracellular lipid trafficking rather than deficiency of a GSL-degrading enzyme, yet miglustat has demonstrated clinically relevant neurological effects [13,14]. MLIV and CLN5 disease further suggest that secondary disturbances of lipid metabolism may influence neurological phenotypes even when the primary molecular defect lies elsewhere [27,33].

This apparent paradox suggests that the decisive variable is not simply whether GSLs accumulate, but whether a miglustat-sensitive pathway remains an upstream and pharmacologically modifiable determinant of ongoing neuronal injury.

The timing of treatment is likely to be critical. Lysosomal storage initiates downstream processes including impaired autophagy, mitochondrial dysfunction, oxidative stress, calcium dysregulation, neuroinflammation, synaptic abnormalities, axonal degeneration, and ultimately neuronal death. Once these processes become self-sustaining, reducing further substrate accumulation may have limited effects on neurological function.

This may help explain the discrepancy between strong responses in young experimental animals and disappointing outcomes in symptomatic human patients. It also provides a plausible explanation for the apparently more favorable signals in some juvenile GM1 patients treated relatively early [7,8].

Another major issue is the distinction between drug failure and target failure. Miglustat is an early-generation iminosugar with relatively broad pharmacology and a therapeutic exposure constrained by gastrointestinal intolerance, weight loss, tremor, and other adverse effects. Therefore, a negative miglustat trial demonstrates that the tested drug regimen was ineffective; it does not necessarily demonstrate that stronger or more selective CNS GCS inhibition would fail.

The MPS III study illustrates this distinction particularly well. Miglustat reached the CNS but did not significantly reduce ganglioside levels [26]. Thus, the trial did not provide unequivocal evidence that effective suppression of CNS GSL synthesis is therapeutically irrelevant; rather, it showed that clinically achievable miglustat exposure did not produce sufficient biochemical or clinical effects.

Hence, further studies should incorporate pharmacodynamic biomarkers capable of confirming CNS target engagement. Without such biomarkers, a negative clinical outcome cannot reliably distinguish inadequate drug exposure from genuine biological failure of the target.

## 14. Miglustat Within the Evolving Therapeutic Landscape

The use of miglustat should be interpreted within a rapidly changing therapeutic landscape. Gene replacement and gene-editing strategies seek to correct the primary molecular defect, whereas intrathecal or intracerebroventricular enzyme delivery aims to overcome the limited CNS exposure of intravenous enzyme replacement therapy. Pharmacological chaperones may increase residual activity of amenable mutant enzymes, while newer brain-penetrant GCS inhibitors may achieve stronger or more selective target engagement than miglustat. These approaches are not interchangeable: their feasibility depends on the affected protein, residual function, disease distribution, treatment timing, and ability to reach vulnerable CNS cell populations. Therefore, comparative interpretation should distinguish failure of a particular compound or delivery route from failure of the broader therapeutic mechanism [6,34].

## 15. Implications for Future Repurposing

Future attempts to repurpose miglustat or related brain-penetrant GSL-modifying compounds should be guided by disease biology rather than by the presence of neurological involvement alone. Modern lipidomic profiling may help identify disorders in which GSL abnormalities are sufficiently prominent and mechanistically linked to neuronal dysfunction to justify therapeutic intervention.

Patient-derived induced pluripotent stem cell models, neurons, cerebral organoids, and disease-specific animal models may further allow pharmacological target engagement to be demonstrated before clinical exposure. Such experiments are particularly important in diseases characterized by secondary lipid abnormalities, where the distinction between pathogenic driver and biochemical epiphenomenon is difficult to establish.

Clinical development should also increasingly focus on early treatment. Improvements in genomic diagnosis, family screening, and newborn screening may allow intervention before substantial neuronal loss has occurred. This may be particularly important for therapies such as SRT that reduce ongoing pathological flux but cannot restore neurons already destroyed by disease.

Combination approaches may ultimately prove more effective than substrate reduction alone. Reducing the generation of new storage material could complement therapies that increase lysosomal clearance or restore the underlying molecular defect. Thus, combinations of SRT with gene therapy, ERT, pharmacological chaperones, or interventions targeting neuroinflammation warrant consideration.

Finally, newer brain-penetrant inhibitors may help determine whether some historical failures represent limitations of miglustat rather than invalidation of the underlying therapeutic concept. The experience with miglustat should consequently be regarded not only as evidence about one drug, but as a framework for the development of future CNS-directed metabolic therapies.

This perspective also keeps the review anchored in the original substrate-reduction concept and early iminosugar pharmacology, while emphasizing that modern repurposing requires disease-specific pharmacodynamic markers and clinically meaningful neurological endpoints [3,5].

## 16. Conclusions

Rare diseases are recognized as a severe medical and social problem, especially due to their high number (thus resulting in large total number of patients despite each disease including a low number of affected persons), severe symptoms, and a lack of effective treatment for most of them [35]. LSD belongs to this group of disorders; therefore, developing effective treatment methods is especially valuable. Miglustat provides a unique pharmacological experiment in the treatment of neuronopathic lysosomal storage disorders. Its ability to reach the CNS and alter GSL metabolism created one of the earliest opportunities to test systemic small-molecule substrate reduction against lysosomal neurodegeneration.

The accumulated evidence demonstrates both the potential and the limitations of this strategy. NPC provides a proof that modulation of GSL metabolism can modify neurological disease even when GSL accumulation is secondary to the primary molecular defect [13,14]. Conversely, controlled studies in GD3, late-onset Tay–Sachs disease, and MPS III demonstrated that BBB penetration and compelling biological rationale do not guarantee neurological efficacy [4,22,26].

GM1 gangliosidosis remains an attractive but unproven candidate for a disease to be treated by miglustat, supported by direct metabolic rationale and encouraging uncontrolled clinical observations [7,8]. Sandhoff disease combines strong experimental evidence with anecdotal human experience [20,21,23]. ML IV demonstrates that manipulation of secondary GSL abnormalities can modify neurodegeneration experimentally even when the primary defect involves a lysosomal membrane protein [27]. CLN3 disease provides an emerging human signal that warrants controlled investigation [27,28,29], while CLN5 disease extends the concept of miglustat-responsive lipid dysregulation to another NCL at the preclinical level [33].

These observations suggest that therapeutic responsiveness cannot be predicted simply from the identity of the primary stored substrate. Rather, successful repurposing is likely to require a miglustat-sensitive lipid pathway that remains sufficiently upstream, pharmacologically accessible, and causally important to ongoing neuronal dysfunction at the time treatment is initiated.

Therefore, the most relevant question is no longer simply whether miglustat crosses the BBB or whether GSL abnormalities are present; it is whether therapeutically achievable CNS exposure can sufficiently modify a disease-driving lipid pathway before downstream neurodegeneration becomes irreversible.

Equally important is the distinction between failure of miglustat and failure of the therapeutic target itself. Further studies, integrating CNS pharmacokinetics, lipidomics, pharmacodynamic biomarkers, natural-history modeling, and earlier intervention, are required to resolve this dilemma and to determine whether the therapeutic lessons learned from miglustat can guide the next generation of brain-directed metabolic therapies for lysosomal neurodegeneration.

## Figures and Tables

**Table 1:** Summary of disease-specific evidence for miglustat in neuronopathic lysosomal storage disorders.

Disorder	Clinical Evidence Level	Controlled Trial Result	Human Evidence Summary	Clinical Implication/Status
Niemann–Pick disease type C	Controlled trial plus longitudinal evidence	Favorable signals in juvenile/adult disease; no demonstrated benefit in early-infantile disease	Strongest human evidence, but phenotype-dependent	Authorized for neurological NPC in some regions; reassess benefit
Gaucher disease type 3	Randomized phase II study	No significant benefit on principal neurological outcomes	Controlled negative evidence	Not authorized for neurological GD3
GM1 gangliosidosis	Small uncontrolled series	No controlled trial	Hypothesis-generating only	Off-label/investigational; controlled study required
GM2 gangliosidoses	Controlled study in late-onset Tay–Sachs disease; anecdotal Sandhoff data	No benefit in late-onset Tay–Sachs study	Negative for tested regimen; target failure not established	Off-label/investigational
Mucopolysaccharidosis type III	Randomized placebo-controlled study	No clinical benefit; CNS ganglioside reduction not demonstrated	Controlled negative evidence	Off-label/investigational
Mucolipidosis type IV	Mouse model only	No human controlled trial	Preclinical evidence only	Investigational
*CLN3* disease	Small open-label study and two-patient report	No controlled trial	Preliminary, hypothesis-generating signal	Off-label/investigational; no efficacy conclusion
*CLN5* disease	Cellular and zebrafish models only	No human controlled trial	Preclinical evidence only	Investigational

## Data Availability

No new data were created or analyzed in this study. Data sharing is not applicable to this article.

## Abbreviations

BBB	blood–brain barrier
CNS	central nervous system
CSF	cerebrospinal fluid
ERT	enzyme replacement therapy
GCS	glucosylceramide synthase
GSL	glycosphingolipid
LSD	lysosomal storage disorder
MLIV	mucolipidosis type IV
MPS III	mucopolysaccharidosis type III
NCL	neuronal ceroid lipofuscinosis
NPC	Niemann–Pick disease type C
SRT	substrate reduction therapy
UBDRS	Unified Batten Disease Rating Scale
